# Time independent factors that predict relapse in adults with acute myeloid leukemia

**DOI:** 10.1038/s41408-023-00954-z

**Published:** 2024-01-15

**Authors:** John J. Lim, Megan Othus, Carole M. Shaw, Kathryn Russell, Anna B. Halpern, Jacob S. Appelbaum, Paul Hendrie, Roland B. Walter, Elihu H. Estey, Mary-Elizabeth M. Percival

**Affiliations:** 1 University of Washington Medical Center, Seattle, WA USA; 2 Public Health Sciences Division, Fred Hutchinson Cancer Center, Seattle, WA USA; 3 Clinical Research Division, Fred Hutchinson Cancer Center, Seattle, WA USA; 4 Division of Hematology and Oncology, Department of Medicine, University of Washington, Seattle, WA USA

**Keywords:** Epidemiology, Acute myeloid leukaemia

Relapse remains common in adults with acute myeloid leukemia (AML), with long-term disease-free survival estimated at 30–40% for patients 60 years or younger treated with chemotherapy [1]. Pretreatment factors, including age, antecedent hematologic disorder (AHD), prior exposure to chemotherapy, karyotype, and molecular mutations, have been used for the prediction of post-treatment outcomes [2, 3].

It has been previously shown that AML patients who achieve initial complete remission (CR) are unlikely to relapse after three years of ongoing CR [4]. In an analysis of over 1000 patients in the first CR, treatment failure was found to be closely related to adverse cytogenetics and older age. Since this prior work, the assessment of treatment response now includes additional factors, including the type of remission (CR vs. CR with incomplete count recovery [CRi]), time to count recovery (if achieved), and presence of measurable residual disease (MRD) [5–7].

We set out to examine the impact of quality of remission, time to count recovery, and presence of MRD in addition to previously identified factors on relapse or death among patients with AML or other high-grade myeloid neoplasms up to 3 years after initial CR. Using our institutional database, we retrospectively identified 972 adults who had a confirmed diagnosis of AML or other high-grade myeloid neoplasm (≥10% blasts in blood or marrow) and underwent initial induction treatment at the University of Washington (UW)/Fred Hutchinson Cancer Center between November 2008 and December 2018. The data cutoff was April 5, 2022. This study was approved by the UW Institutional Review Board.

Disease status was defined as de novo vs. secondary, with secondary including AHD (antecedent myelodysplastic syndrome or myeloproliferative neoplasm) or therapy-related. Induction chemotherapy was divided into three groups: high intensity included multiagent chemotherapy with cytarabine at ≥1 g/m^2^/dose/day (such as CLAG-M [8], FLAG-Ida, or similar); intermediate intensity included 7 + 3 or similar; and low intensity was defined as a hypomethylating agent with or without venetoclax. MRD was quantified using multiparameter flow cytometry and detected once a patient achieved morphologic CR or CRi with <5% blasts in the marrow; MRD was measured at a single time point, usually around day 30 after induction, and was not reassessed later. MRD assessment was performed on marrow using multiparameter flow cytometry with a minimum sensitivity of 0.1% [9]. Relapse was considered to have occurred if the bone marrow contained ≥5% blasts or if circulating peripheral blasts were identified.

The primary outcomes were binary endpoints of survival without relapse (relapse-free survival, RFS) at 1, 2, or 3 years after the achievement of morphologic CR following initial therapy. Patients without relapse and censored before the landmark dates were excluded from the respective analyses. Multivariable logistic regression models were used to evaluate covariate associations with these outcomes; covariates evaluated were: age (assessed as a continuous variable), ELN 2017 risk classification, presence of MRD, gender, WBC count at diagnosis (assessed as a continuous variable), secondary disease, ECOG performance status (0–1 vs. 2–4), treatment-related mortality (TRM) score [10], induction intensity, CR vs. CRi, early blood count recovery (defined as absolute neutrophil count (ANC) ≥ 1000/µL and platelet count ≥ 100,000/µL within 30 days), and transplantation status. The Kaplan-Meier method was used to estimate RFS and overall survival. All analyses were performed using R version 4.2.2.

In all, 656 patients achieved a morphologic remission (CR or CRi) defined by European LeukemiaNet (ELN) 2017 criteria [11] and were included in our analysis. The characteristics of the 656-patient cohort are summarized in Table 1. The median age was 60, with a range of 18–91 years. Three hundred seventy-three patients (57%) had de novo AML, and 283 (43%) were defined as secondary due to prior chemotherapy and/or AHD. Pretreatment molecular and cytogenetic information was available for all but 6 patients, and 72% were classified as intermediate or adverse by ELN 2017 criteria. For patients that did not have all data points to classify risk according to ELN, clinical assessment at the time of diagnosis, as well as available data (e.g., molecular markers, cytogenetics/FISH), were used. The majority of patients received high- or intermediate-intensity induction (88%), and most (82%) patients had an ECOG performance status of 0–1 at diagnosis. Five hundred forty (82%) patients achieved a CR (<5% marrow blasts with peripheral count recovery). MRD was identified in 173 patients (26%). Two hundred and seventy patients (41%) received subsequent allogeneic hematopoietic cell transplant (HCT). Median follow-up among all patients was 5.2 years (range 1 month to 13.2 years).

**Table 1:** Characteristics of the 656-patient cohort.

Characteristic	Value
Age, median (range)	60 (18–91)
*Gender, no. (%)*	
Male	374 (57%)
Female	282 (43%)
*ELN 2017 risk group, no. (%)*	
Favorable	181 (28%)
Intermediate	266 (41%)
Adverse	203 (31%)
NA	6 (1%)
* WBC at diagnosis, median (range, cells/µL)*	7160 (100–586 500)
*Performance status, no. (%)*	
0–1	538 (82%)
2–4	118 (18%)
*Disease status, no. (%)*	
De novo	373 (57%)
Secondary	283 (43%)
*Induction intensity, no. (%)*	
High	417 (64%)
Intermediate	160 (24%)
Low	79 (12%)
*Remission status, no. (%)*	
CR	540 (82%)
CRi	116 (18%)
*MRD status, no. (%)*	
MRD−	483 (74%)
MRD+	173 (26%)
*Treatment response, no. (%)*	
CR MRD−	416 (63%)
CR MRD+	124 (19%)
CRi MRD−	67 (10%)
CRi MRD+	49 (7%)
*Count recovery, no. (%)*	
Within 30 days	235 (36%)
>30 days or no recovery	421 (64%)
* Subsequent allogeneic HCT, no. (%)*	270 (41%)
*Alive without relapse*	
Year 1	347
Year 2	265
Year 3	228

Abbreviations: *ELN* European LeukemiaNet, *WBC* white blood cell, *NA* not available, *MRD* measurable residual disease, *CR* complete remission, *CRi* complete remission with incomplete count recovery, *HCT* hematopoietic cell transplantation.

Older age was significantly associated with decreased RFS and was significant at years 2 and 3 of the landmark analyses [year 2 OR 1.19, 95% CI 1.03–1.37; year 3 OR 1.17, 95% CI 1.01–1.36; Table 2). ELN 2017 intermediate (year 1 OR 3.77, 95% CI 2.22–6.4; year 2 OR 3.49, 95% CI 2.05–5.94; year 3 OR 3.43, 95% CI 1.95–6.04) and adverse (year 1 OR 5, 95% CI 2.76–9.03; year 2 OR 3.63, 95% CI 2–6.58; year 3 OR 3.23, 95% CI 1.73–6.06) risk groups were significantly associated with increased risk of relapse or death and remained significant at each of the three landmark analyses. Certain patient characteristics were associated with significantly higher odds of relapse or death by years 1 and 2, including elevated WBC (year 1 OR 2.84, 95% CI 1.41–5.74, year 2 OR 2.65, 95% CI 1.27–5.54) and those with higher PS (year 1 OR 2.99, 95% CI 1.59–5.63; year 2 OR 1.95, 95% CI 1.02–3.76). However, these factors lost significance in the year 3 analysis. Of all patients that relapsed or died in the first year, 83% (238) were intermediate or adverse risk using ELN 2017 criteria.

**Table 2:** Multivariable logistic regression to evaluate the outcome of relapse at years 1, 2, and 3 after initial complete remission (CR).

	Year 1 (*n* = 625)		Year 2 (*n* = 612)		Year 3 (*n* = 602)	
Covariate	OR, 95% CI	*p*-value	OR, 95% CI	*p*-value	OR, 95% CI	*p*-value
* Age (per 10 years)*	1.14, 0.99–1.32	0.077	1.19, 1.03–1.37	0.017	1.17, 1.01–1.36	0.038
* Male (ref* *=* *female)*	1.15, 0.77–1.71	0.48	1.26, 0.85–1.87	0.26	1.36, 0.9–2.05	0.14
* Elevated WBC (ref* *=* *WBC* *<* *100,000/µL)*	2.84, 1.41–5.74	0.0036	2.65, 1.27–5.54	0.0096	1.84, 0.85–3.99	0.12
*Secondary disease*^a^						
Prior AHD	1.61, 0.99–2.61	0.055	1.31, 0.8–2.13	0.28	1.04, 0.63–1.72	0.88
Prior AHD and therapy-related	1.75, 0.76–4.02	0.19	0.99, 0.42–2.32	0.98	0.79, 0.32–1.94	0.61
Therapy-related	0.58, 0.25–1.34	0.2	0.68, 0.3–1.53	0.35	0.81, 0.35–1.88	0.63
* ECOG PS 2–4 (ref* *=* *PS 0–1)*	2.99, 1.59–5.63	<0.001	1.95, 1.02–3.76	0.044	1.06, 0.52–2.15	0.87
* TRM score*	0.99, 0.97–1.02	0.67	1, 0.97–1.03	0.84	1.06, 1.01–1.11	0.018
*ELN 2017 risk group*^b^						
Intermediate	3.77, 2.22–6.4	<0.001	3.49, 2.05–5.94	<0.001	3.43, 1.95–6.04	<0.001
Adverse	5, 2.76–9.03	<0.001	3.63, 2–6.58	<0.001	3.23, 1.73–6.06	<0.001
*Treatment intensity*^c^						
Intermediate intensity	0.97, 0.6–1.59	0.92	0.95, 0.58–1.54	0.83	0.67, 0.4–1.1	0.11
Low Intensity	0.44, 0.23–0.87	0.018	0.72, 0.36–1.44	0.35	0.79, 0.37–1.67	0.53
*Treatment response*^d^						
CR MRD+	6.99, 4.04–12.1	<0.001	7.87, 4.34–14.28	<0.001	7.91, 4.14–15.13	<0.001
CRi MRD−	1.84, 0.95–3.54	0.069	1.93, 0.96–3.9	0.065	2.05, 0.96–4.39	0.064
CRi MRD+	2.26, 1.04–4.91	0.039	2.1, 0.93–4.71	0.073	2.48, 1.06–5.82	0.021
* Count recovery* *>* *30 days or no recovery (ref* *=* *count recovery within 30 days)*	1.43, 0.93–2.19	0.11	1.26, 0.82–1.92	0.3	1.29, 0.83–2.01	0.25
* Transplant* *(ref* *=* *no transplant within 1–3 years)*	0.13, 0.08–0.21	<0.001	0.13, 0.08–0.21	<0.001	0.13, 0.08–0.22	<0.001

Abbreviations: *ref* reference, *OR* odds ratio, *WBC* white blood cell, *AHD* antecedent hematologic disorder, *PS* performance status, *TRM* treatment-related mortality, *CR* complete remission, *CRi* complete remission with incomplete count recovery, MRD measurable residual disease.

^a^Reference group for all comparisons = de novo.

^b^Reference group for all comparisons = favorable risk.

^c^Reference group for all comparisons = high intensity.

^d^Reference group for all comparisons = CR MRD−.

In the models summarized in Table 2, treatment response (CR with MRD vs. CR without MRD) had an association for relapse or death at each time point tested (year 1 OR 6.99, 95% CI 4.04–12.1; year 2 OR 7.87, 95% CI 4.34–14.28; year 3 OR 7.91, 95% CI 4.14–15.13). MRD status was also significantly associated with RFS and OS (RFS: MRDneg 47%, 95% CI 43–52%, MRDpos 19%, 95% CI 14–26%; OS: MRDneg 56%, 95% CI 52–61%, MRDpos 28%, 95% CI 22–36%). Other covariates had an initial significant OR for relapse or death at years 1 and 2 but this finding was lost over time. For example, elevated WBC carried a significant risk for relapse or death through years 1 and 2 but lost significance at year 3. Additionally, higher performance status (ECOG 2–4) conferred an increased risk of relapse or death at years 1 and 2 but was not significant at year 3. The evidence of an association between time to count recovery and outcome was less strong (year 1 OR 1.49, 95% CI 1–2.23; year 2 OR 1.32, 95% CI 0.89–1.97; year 3 OR 1.35 95% CI 0.89–2.03). Treatment intensity was not a significant predictor of RFS in multivariable models at any timepoint. Receipt of HCT was significantly associated with a decreased risk of RFS in all models.

In our analysis of 656 patients, the strongest and most consistent predictors of RFS were treatment response (MRD vs. no MRD for patients in CR) and ELN 2017 risk since they remained significant at all three time points examined (year 1, year 2, and year 3). Increased age and incomplete count recovery were also associated with relapse or death, though not significant at every time point. Similarly, other factors, including ECOG performance status and high WBC count at diagnosis, were associated with relapse or death in years 1 and 2 but not year 3.

These results stress the importance of post-therapy data (e.g., MRD status) in prognostication of outcomes after completing initial therapy. Therapy intensity was not associated with RFS in multivariable models, though notably, the majority of patients received high (64%) or intermediate (24%) intensity induction. Recent data published by Bazinet and co-authors suggest that the goal of any AML-directed treatment should be MRD-negative CR [12]. The findings in our study that the presence of MRD was significantly associated with RFS—but therapy intensity was not—would also support this conclusion.

Our strengths include the large number of patients included in our study with substantial follow-up, which allowed us to evaluate the impact of a number of covariates in multivariable analyses. Furthermore, our population was heterogeneous, with various treatment regimens reflecting the current treatment paradigm of the disease. Our study was limited in that it is retrospective in nature and performed at a single center.

The major goal of our study was to evaluate the prognostic importance of several covariates for the outcomes of relapse and non-relapse death through years 1–3 following initial CR for patients with AML and other high-grade myeloid neoplasms. Based on our findings, patients who are older, MRD positive, intermediate or adverse cytogenetic risk, or demonstrate incomplete recovery of their peripheral counts have an ongoing increased risk of relapse or death in the first 3 years. Future studies should evaluate strategies to treat patients with MRD since prognosis is poor and effective treatment options are limited. Other unanswered questions include evaluating the ideal number of intensive consolidation cycles, the role of maintenance chemotherapy, and the benefit of allogeneic HCT, particularly in older patients.
